# *Pythium insidiosum* keratitis reported in China, raising the alertness to this fungus-like infection: a case series

**DOI:** 10.1186/s13256-021-03189-3

**Published:** 2021-12-17

**Authors:** Hongyan Hou, Yue Wang, Lei Tian, Feng Wang, Ziyong Sun, Zhongju Chen

**Affiliations:** Department of Laboratory Medicine, Tongji Hospital, Tongji Medical College, Huazhong University of Science and Technology, Jiefang Road 1095#, Wuhan, 430030 China

**Keywords:** *Pythium insidiosum*, Keratitis, rRNA gene sequencing, MALDI-TOF, Mass spectrometry

## Abstract

**Background:**

The objective of this study is to report typical clinical and laboratory characteristics of three cases of keratitis caused by *Pythium insidiosum* in China.

**Case presentation:**

Three Chinese patients of Han nationality diagnosed with* Pythium* keratitis from 2017 to 2019 were included. One 45-year-old female and one 55-year-old male were exposed to river water, and one 51-year-old female was burned by ash in the eyes. All of them are of Han ethnicity. Upon slit-lamp examination, subepithelial and superficial stromal opacities were observed in a reticular pattern. After conventional treatment with antifungal agents, the clinical status worsened and therapeutic penetrating keratoplasty was performed. Unfortunately, enucleation was performed to remove all infected tissue and relieve pain. *Pythium insidiosum* was identified in culture and confirmed by internal transcribed spacer ribosomal RNA gene sequencing analysis. Following the systemic and local antibiotic regimens, the patients were cured ultimately and no regression of infection was observed.

**Conclusions:**

It is significant for ophthalmologists and microbiologist to be alert to this eye-threatening infection, especially in patients who are resistant to antifungal treatments and with water-related exposure.

## Background

*Pythium insidiosum* is a genus of aquatic fungal-like oomycete pathogen belonging to the kingdom Stramenopila [1]. *P. insidiosum* is often found in tropical, subtropical, and temperate climates [2]. Human pythiosis is a rarely occurring disease and has four major presentations: vascular, ocular, cutaneous/subcutaneous, and disseminated infection [1]. Vascular pythiosis is the most common manifestation in thalassemia patients with high morbidity and mortality [1, 3]. Ocular infection caused by *P. insidiosum* usually occurs in healthy people and poses a threat to the eyes [1, 4]. Possible risk factors for *P. insidiosum* keratitis include trauma, stagnant water exposure, and contact lens wear [5–8]. *P. insidiosum* keratitis has been reported in several countries, including Thailand, India, China, Australia, Haiti, and Israel [9]. Thailand has the highest percentage of pythiosis, and the rate of enucleation is up to 55–79% in these patients [1, 7]. Ocular infections caused by *P. insidiosum* have been found to be under-recognized due to the rarity and limited knowledge of the disease.

*P. insidiosum* keratitis is often misdiagnosed as fungus keratitis because they have similar clinical presentation and morphology in diagnostic scrapings. Accurate microbiological identification is based on zoospore induction. Nowadays, DNA sequencing can be used for its identification [10]. *P. insidiosum* keratitis is recalcitrant to antifungal therapy because it lacks ergosterol in its cytoplasmic membrane [11]. As the conventional antifungal agents are ineffective, most patients require therapeutic keratoplasty and/or eventual enucleation [4]. Currently, there is no standard protocol for *P. insidiosum* keratitis, and combination of antibacterial medications has been proven to be an effective therapeutic regimen.

Although several cases of *P. insidiosum* keratitis have been reported worldwide, only one case of pythiosis originated from China, occurring in a 7-year-old child [9]. Here, we present three cases of *P. insidiosum* keratitis in adults from China that progressed to enucleation to control the infection despite intensive medical and surgical therapy.

## Case presentation

### Case 1

**Fig. 1:**
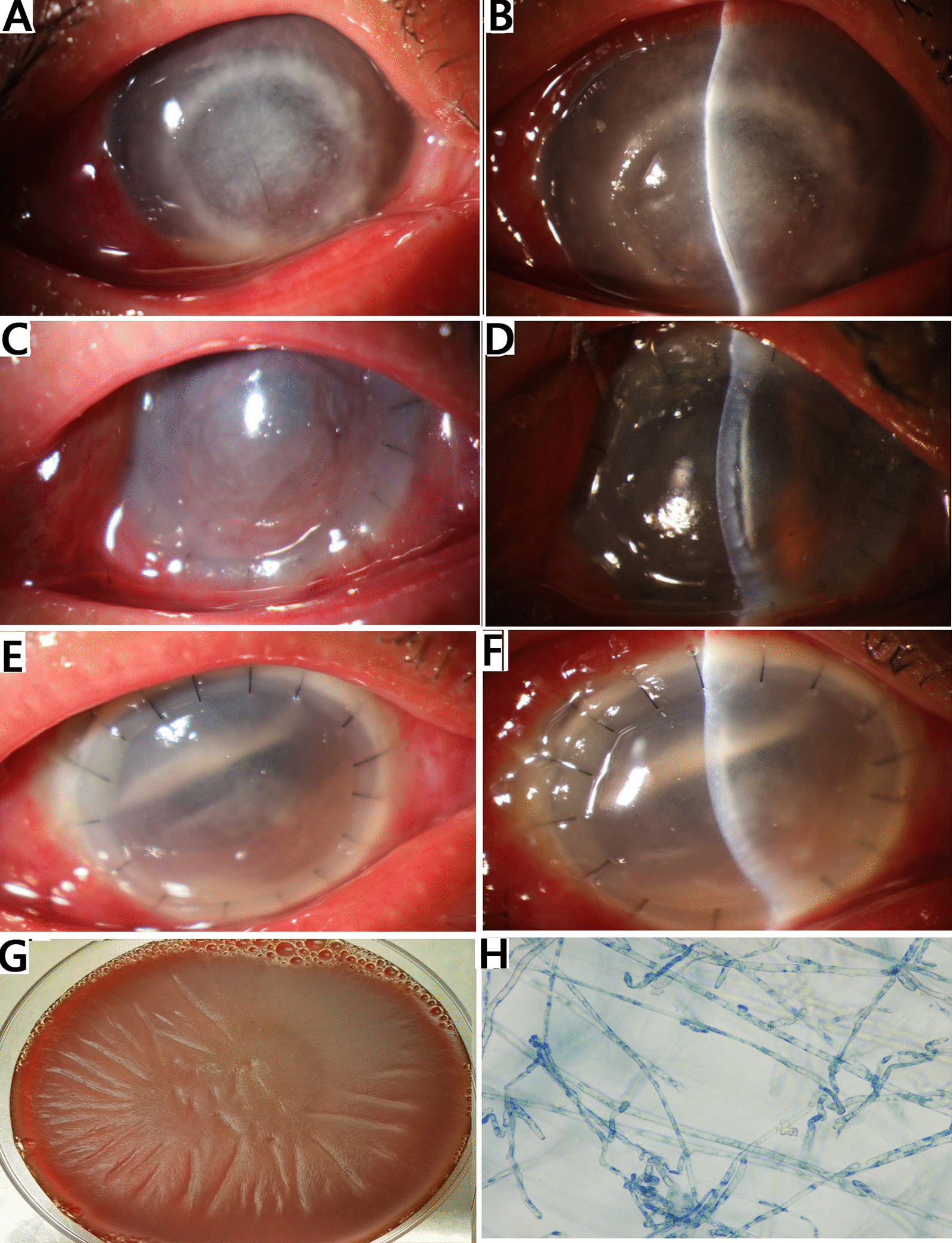
Slit-lamp and direct microscopy digital images. **A**, **B** Photographs demonstrating that right eye showed a central corneal ulcer with a diameter of about 6 × 6 mm with underlying dense stromal infiltrates. **C**, **D** Day 1 following therapeutic penetrating keratoplasty, hyperemia and a thin exudation membrane in the anterior chamber were discernible. **E**, **F** Day 4 postsurgery, central infiltrate with superficial radiating reticular pattern with hypopyon was visible. **G** Microbial culture of *P. insidiosum* collected from corneal tissue at 37 °C grown on 5% sheep blood agar. **H** *P. insidiosum* stained with lactophenol blue revealing branching filamentous fungus-like hyphae (original magnification ×400)

A 45-year-old female of Han nationality presented to a cirneal clinic in August, 2017 with a history of pain, redness, and decreased vision in her right eye 1 week after being exposed to river water. Corneal scrapings and confocal microscopy *in vivo* were performed instantly. A mass of hyphae was found in the 10% KOH wet mount stained with lactophenol blue and examined via confocal microscopy. Fungal keratitis was identified, and right corneal keratectomy was performed. Empirical antifungal and antibacterial therapy was initiated including topical and systemic fluconazole, levofloxacin, and cefminox sodium. After treatment of 2 weeks, the ulcer and symptoms did not improve and the patient was admitted to our hospital. Conjunctival congestion persisted, and a central corneal ulcer with a diameter of around 6 mm that reaches deep into the stromal layer can be seen (Fig. 1A, B). Subepithelial and superficial stromal opacities with dot-like and tentacle-like infiltrates accompanied this. Intracameral fluconazole injection was used to conduct a lamellar keratoplasty. Amphotericin B was administered immediately after the surgery. The first day post-surgery, hyperemia and a thin exudation membrane in the anterior chamber were discernible (Fig. 1C, D). Four days after the keratoplasty, full-thickness large central infiltrate with hypopyon was observed in the right eye (Fig. 1E, F). Given the increasing infiltrate with hypopyon and ineffectiveness of antifungal therapy, the right eye was enucleated. Microbial culture for corneal tissue revealed fungus-like organism showing long sparsely septate hyaline hyphae (Fig. 1G, H). The organism was further identified as *P. insidiosum* by ribosomal RNA (rRNA) gene sequencing with panfungal primers (ITS1/ITS4), which matched 99.23% with the *P. insidiosum* strain (GU137348.1). Then, the patient was adjusted with combined antibacterial treatment consisting of linezolid and azithromycin, and no recurrence of infection was observed on follow-up.

### Case 2

**Fig. 2:**
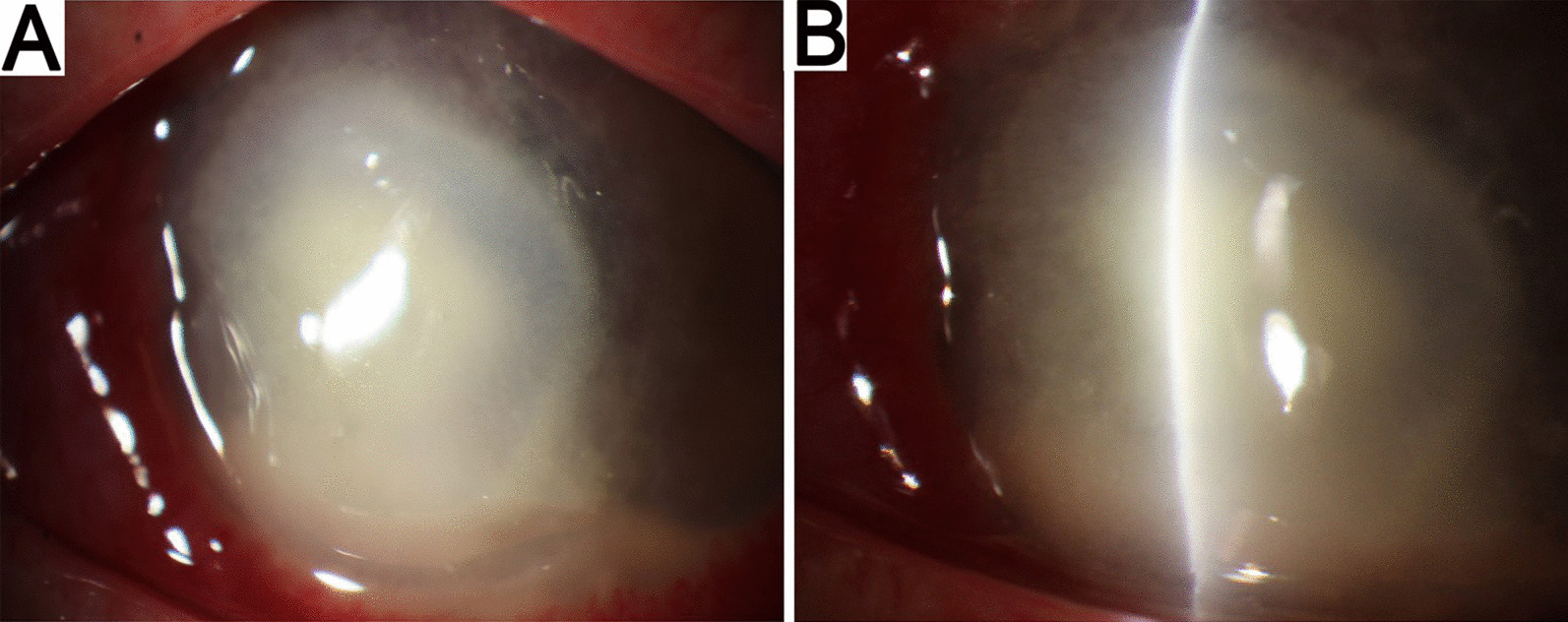
Slit-lamp picture of the cornea. **A**, **B** Large dense grayish-white infiltrate emanating from the main infiltrate extending to the peripheral cornea

**Fig. 3:**
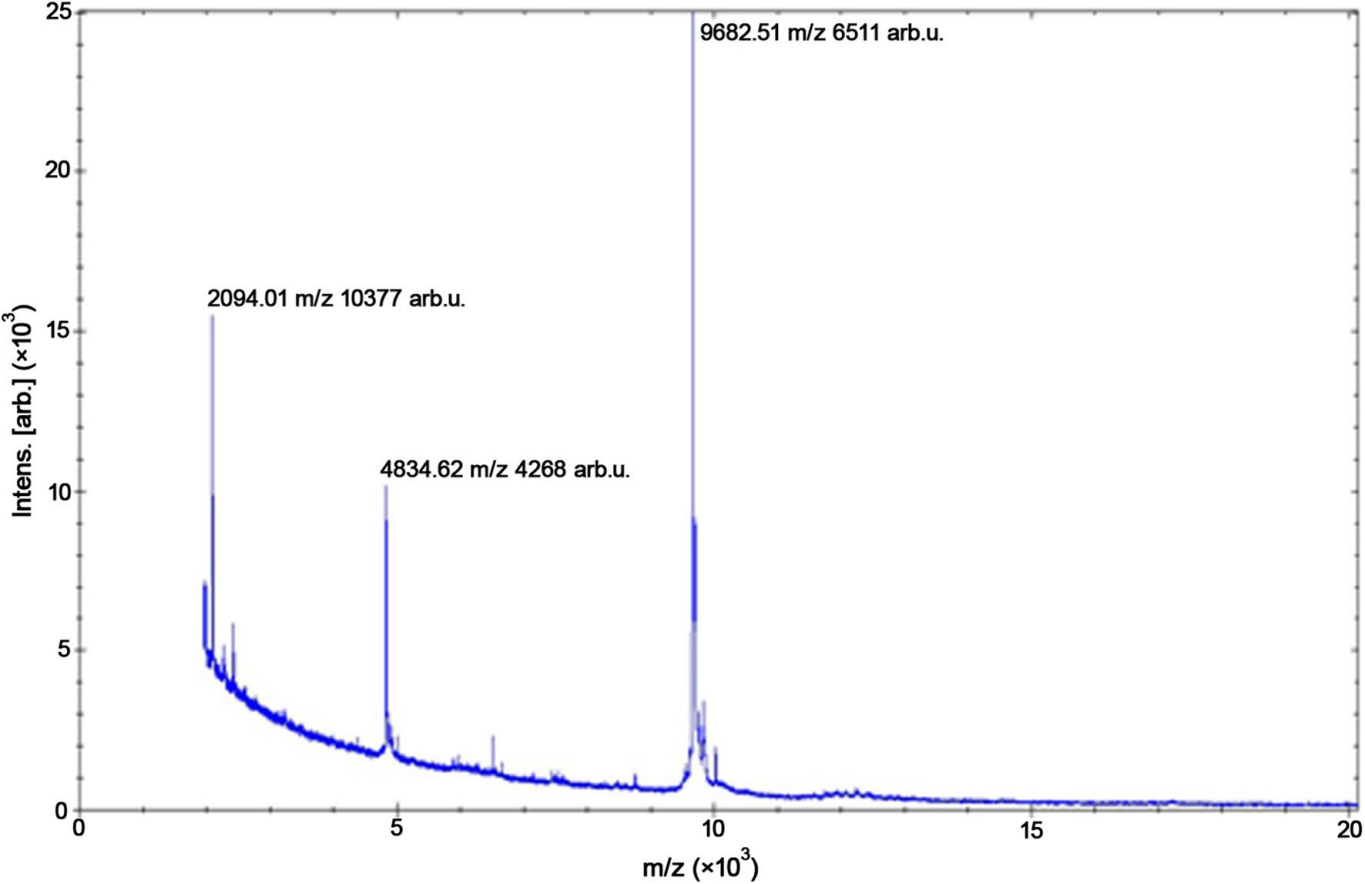
MALDI-TOF-MS image. Three obvious protein peaks of *P. insidiosum* were identified by MALDI-TOF-MS

A 51-year-old female of Han nationality was referred to our hospital in September 2018 with complications of pain, redness, and hyperemia in her right eye after entry of some cigarette ash 1 week ago. Corneal scraping was performed, and Gram staining, KOH preparation, and cultures were negative. Antifungal and antibacterial treatment was initiated including levofloxacin eye drops, cefminox sodium, and voriconazole. After 2 weeks of outpatient treatment, the patient was hospitalized. A grayish-white ulcer was observed in the central of bitamporal cornea measuring 4 × 6 mm. Inflammatory infiltrates with feathered margins and hypopyon with a depth of 2 mm were seen, suggestive of a fungal infection. The cornea showed dense central stromal opacity surrounded by a reticular pattern of subepithelial and superficial stromal infiltration (Fig. 2). Then penetrating keratoplasty was performed. Exudation was observed in anterior chamber on 2 days following surgery, and intracameral fluconazole injection was performed. However, the infiltrates extended progressively, and were unresponsive to any treatment. Therefore, by day 28 post-exposure, an enucleation was performed to remove infected tissue and relieve pain. One week later, a small amount of mycelial growth was observed within the corneal fragment on the potato dextrose agar (PDA) plate. Subculture in brain–heart infusion resulted in the rapid growth of a large mycelium at 35 °C. We have attempted to identify this mycelium by matrix-assisted laser desorption ionization–time of flight mass spectrometry (MALDI-TOF-MS) but failed. There was no reference spectrum in the Bruker Filamentous Fungi databases despite the high quality of the protein spectrum. The mycelium was sent for internal transcribed spacer (ITS) rRNA gene sequencing analysis and unambiguously identified as *P. insidiosum.*. Three obvious protein peaks of the strain were found by MALDI-TOF-MS, which are 2094.01, 4834.62, and 9682.51 (× 103 *m*/*z*), respectively (Fig. 3). Then, the reference spectrum of *P. insidiosum* was added to the in-house database according to Bruker MALDI-TOF-MS standard process to create a library. Therefore, the identification of *P. insidiosum* could be facilitated using MALDI-TOF-MS.

### Case 3

**Fig. 4:**
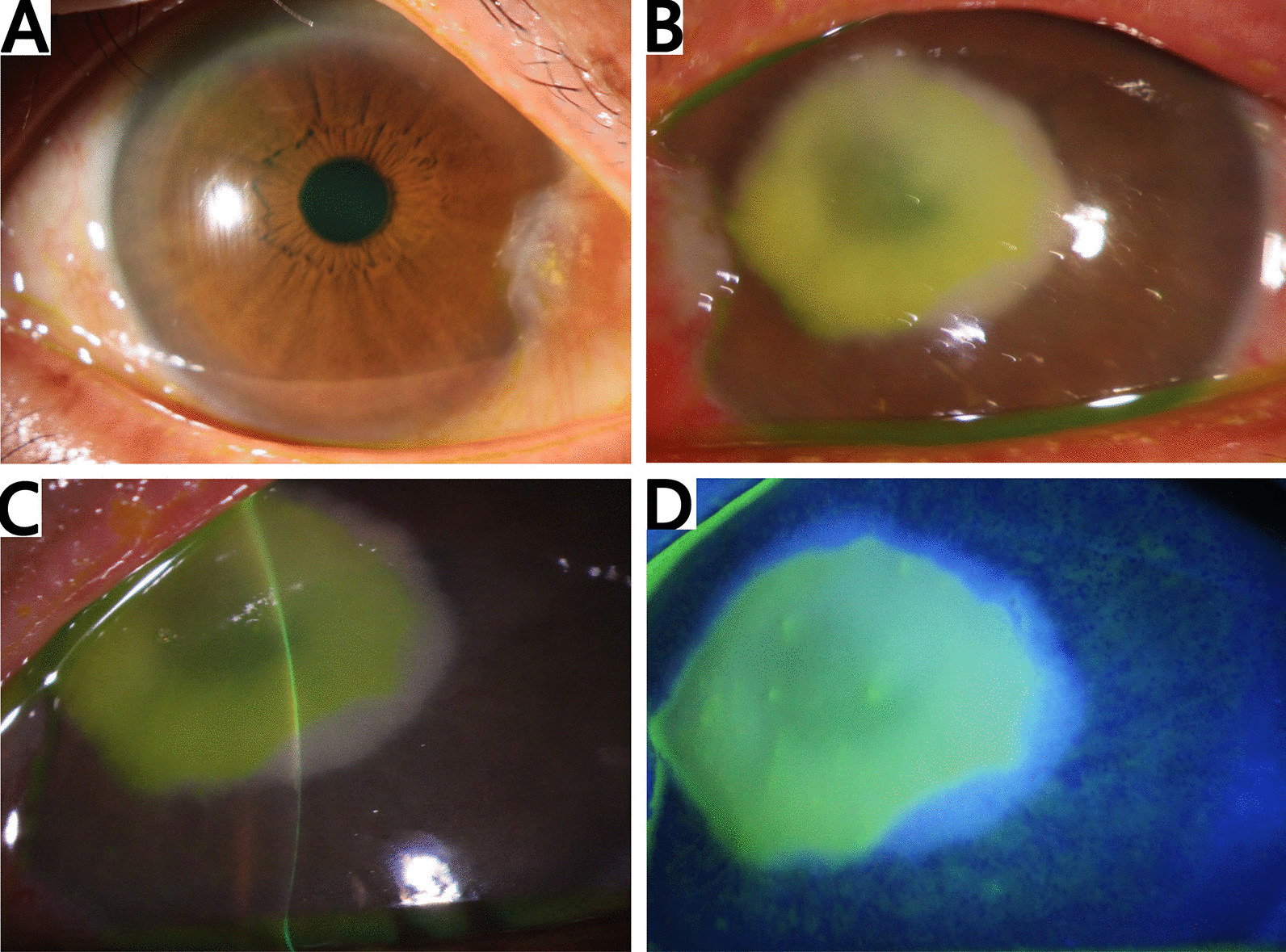
Slit-lamp digital images. **A** Normal right eye. **B**–**D** Significant grayish-white infiltrate of cornea with a diameter of about 5 mm

A 55-year-old male of Han nationality presented with irritation, pain, and hyposia in the left eye for 3 days after facial washing with contaminated river water. He went to a local ophthalmic clinic and was diagnosed with viral keratitis. After 4 days of antiviral therapy, the symptoms were not improved, so the patient was admitted to our hospital. Slit-lamp examination showed conjunctival hypertrophy and infiltrated growth into cornea about 2 mm from the nasal limbus. In the left eye, microvascular tissue hypertrophy of about 2.5 mm was seen across the corneal limbus, and hyperemia grayish-white infiltrate of cornea with a diameter of about 5 mm was observed (Fig. 4). Direct microscopy of corneal scrapings stained with Gram and KOH preparation was negative for organisms. The patient was prescribed empiric fortified topical and systemic antibiotics, including ornidazole, tobramycin, vancomycin, natamycin, and fluconazole. On the third day after hospitalization, symptom improvement was not noted. Excision of pterygium and therapeutic penetrating keratoplasty were performed in the left eye. Cultures of his corneal tissue for bacteria, fungus, and *Acanthamoeba* were negative. Because there was evidence of increased keratoneuritis, antiamebic therapy (chlorhexidine) was initiated and voriconazole was added. Twelve days after the surgery, corneal opacity was rescraped and infiltration extended deeply into the anterior chamber. A second penetrating keratoplasty, virtually limbus to limbus, and intracameral amphotericin B injection were performed sequentially. However, 10 days after the second operation, the infection still spread to the adjacent sclera and progressed to endophthalmitis. Enucleation eventually had to be done. Subsequently, the corneal cultures growing on PDA plate were identified as *P. insidiosum* by MALDI-TOF-MS, confirming the diagnosis of pythiosis.

## Discussion

*P. insidiosum*, oomycete, is a fungal-like organism that develops hyphae similar to those found in true fungi and is found predominantly in aquatic environments. *Pythium* keratitis is a rare but destructive infectious disease, with most cases reported from Thailand [1]. Recently, the large number of cases occurring in India deserved close observation. The researchers demonstrated that ocular pythiosis is underdiagnosed and might be labeled as unidentified fungi. The main reasons for the misdiagnosis of pythiosis include lack of characteristic clinical features, unawareness among clinicians, and a lack of early diagnosis in some regions. Therefore, there is a need for an increase in awareness among both microbiologists and ophthalmologists [4].

Although systemic infection with *P. insidiosum* has been reported in other countries, only one case of *P. insidiosum* keratitis in a 7-year-old child was reported from China [9]. In this report, we firstly described three cases of ocular pythiosis in adults from central China. Two of them presented with corneal keratitis after exposure to river water, and one has trauma in the eye. Previous studies have shown that aquatic environments, particularly contaminated rainwater and/or floodwater, are an important risk factor for the occurrence of *Pythium* keratitis [2, 12, 13]. In addition, contact lenses also have been reported to be implicated with *Pythium* keratitis, but both reported cases also had a history of tap water contamination [5, 14]. The clinical characteristics of our series are similar to those of fungus keratitis, for example, stromal infiltrate with a feathery margin and subepithelial and superficial stromal infiltration in a reticular pattern especially on the border. However, these features of corneal lesions may be concealed by dense stromal infiltration or corneal opacity from toxicity of topical medications. The corneal findings at the first visit may provide important clues for presumptive diagnosis of *Pythium* keratitis.

Currently, the diagnostic tests for *Pythium* have some limitations. Culture identification with zoospore induction is used to confirm the pathogen [15]. However, this test may need 5–7 days and delay the treatment significantly. Confocal microscopy is a noninvasive real-time analysis of causative organisms in microbial keratitis [16]. KOH examination obtained from corneal scraping showed that the typical features of *Pythium* hyphae in a smear have a size of 3–10 μm, sparse septation, and perpendicular lateral branches [2, 12]. Histopathology may be the most useful test providing indications for the presumptive diagnosis of *Pythium* keratitis. However, confocal microscopy, KOH, and histopathology cannot discriminate *Pythium* from true fungal agents. PDA plate is a commonly available medium in most clinics and was used in our cases. The culture has been demonstrated as colorless to white with finely radiated colonies. Serological methods, including immunodiffusion and enzyme-linked immunosorbent assay, developed to detect antimicrobials to *P. insidiosum* have also been used to diagnose pythiosis. The immunological method have been reported to have high specificity, but the sensitivity is low with only 40% [1]. Recently, polymerase chain reaction (PCR) and sequencing have been developed to provide a definitive diagnosis of *P. insidiosum* infection. In the present report, we have created a reference spectrum of *P. insidiosum* by MALDI-TOF-MS after ITRNS rA gene sequencing and added it to the Bruker database, which could be used to facilitate diagnosis of*P. insidiosum* infection in the future. Moreover, we have compared the reference spectrum of *P. insidiosum* in our laboratory with the reported one, which showed high similarity [17]. Therefore, the application of ITS rRNA gene sequencing and MALDI-TOF-MS was time-saving and allowed immediate identification of the pathogen.

The standard protocol for medical management of human pythiosis has not been reported. As *Pythium* is not a fungus, antifungal reagents are not useful. *In vitro* susceptibility of *P. insidiosum* to antibacterials, including minocycline, tetracyclines, macrolides, and linezolid, has been reported and supports the use of antibacterial medications for this infection [18, 19]. Combination of linezolid, azithromycin, and atropine sulfate were reported to be successful in resolution of human *P. insidiosum* keratitis [11]. One new study shows that triple therapy consisting of minocycline, linezolid, and chloramphenicol might be a promising candidate treatment for *P. insidiosum* [20]. In our cases, antifungal agents were used during the progression, and the diagnosis of *P. insidiosum* keratitis was made after enucleation surgery. Therefore, the patients were misdiagnosed and missed the best time for treatment. To our knowledge, this is the second report of *P. insidiosum* keratitis in Chinese and the first one originating from Chinese adults. Due to the rare occurrence of *P. insidiosum* keratitis, practitioners should be alert to this disease in patients exposed to river water and showing no improvement with antifungal therapy.

## Conclusion

*P. insidiosum* keratitis is often misdiagnosed as typical mycotic keratitis, and the use of antifungal therapy is ineffective. Therefore, this etiologic agent should be considered in cases of suspected keratomycosis with water-related exposure, particularly if the infection is resistant to antifungal treatments. The wide application of DNA sequencing and MALDI-TOF-MS methods can facilitate the early diagnosis of the disease. Moreover, pythiosis was not limited to tropical and subtropical areas and might occur widely. Furthermore, new antimicrobials need to be explored to eradicate this intriguing organism in ophthalmic practice.

## Data Availability

All data are contained within the manuscript. Clinical isolates will be made available by ZJC upon request.

## Abbreviations

rRNA: Ribosomal RNA
PDA: Potato dextrose agar
MALDI-TOF MS: Matrix-assisted laser desorption ionization–time of flight mass spectrometry
